# The Council Meeting

**Published:** 1907-08-10

**Authors:** 


### THE COUNCIL MEETING.

### THE ANNUAL REPORT AND GRANTS.

Sir James Ritchie (in the absence of the Lord Mayor) presided at a meeting of the Council of the Metropolitan Hospital Sunday Fund at the Mansion House on Thursday, August 1.

On the motion of Sir William Church, seconded by Mr. Grey, the report of the Committee of Distribution was adopted, and an order made for the payment of the awards.

Mr. John Hampton Hale moved to receive a recommendation of the Finance and General Purposes Committees to accept a proposition of the executors of the late Mr. George Herring that a sum of £30,000 on account of the residuary bequest, being about equivalent to one year's interest theron, be paid over, and that the same be added to the collection for distribution this year. The difficulty was that, the estate being a large one, the legacies had not yet been paid, and the residuary estate could not therefore be distributed. However, the matter had been dealt with in the most friendly manner, and they had been glad to embrace the proposals of the executors as embodied in the resolution. The motion was adopted.

In the absence of Sir John Bell, Mr. Hale asked leave to move the resolution standing on the agenda paper in his name, namely : "That before any hospital or institution receiving a grant from this fund enters into a contract for the provision of additional accommodation for patients, or any extraordinary expenditure which would increase the cost of maintaining the hospital, the consent or approval of the Council of this Fund must be obtained."

Mr. J. Astley Bloxam seconded.

Sir Edmund Hay Currie (the secretary) supported the motion. He said they were aware that at least three hospitals were providing additional accommodation, while it was common knowledge that some thousand beds in London hospitals were unoccupied. He thought it was important that the opinion of the Fund should be expressed in the matter. That was essentially a financial Fund, and did not interfere with the internal administration of the hospitals. But these three hospitals were aiming at providing new beds when there were not enough funds to carry on those already existing in the Metropolis. Two of these hospitals were only just able to hold their heads above water, while in the third the expenditure exceeded the receipts. A thousand beds meant a cost of £100,000 a year to fill and maintain them. One hospital had put up a magnificent building, and had not been able to treat a single patient in the wards; another had spent all its proprietary funds, and its wards stood empty. Still another had borrowed money from its bankers for building, and its funds only sufficed to pay interest on its borrowings. It was quite well known to that Fund, which was in a position to survey the whole field, that if there was not sufficient hospital accommodation in any particular locality there was plenty elsewhere. True the Sunday Fund would not give money for bricks and mortar, but they had to provide money for the maintenance of the extra accommodation. They were bound to consider the state of the finances of the hospitals for the year. The 62 hospitals in London owed among them £150,000 to tradesmen and to bankers for current loans; and £100,000 less had been received for legacies. With one or two notable exceptions the hospitals were doing extremely badly, and Sir John Bell felt that they ought not to let another year go by without representing to the gentlemen responsible, in the most friendly manner, the circumstances of the whole of London. It was almost sinful to go on with extensions with the absolute certainty that money to maintain them would not be forthcoming. So far as could be seen, the Sunday Fund collection would work out at from five to six thousand pounds less than last year. From whatever cause—"week-ending" or whatever it might be—the donations of some of the West-End churches which had worked splendidly for them in the past had gone down; and some of their great donors had not been able to help them so well as formerly. All this ought to be brought home to hospital managers who contemplated extensions.

The Hon. Sydney Holland considered that the proposal involved a great alteration in the policy of the Fund, and that it would, in fact, mean interference with the internal administration. Under it a hospital could not introduce the Finsen light or start a laundry. He did not know where Sir Edmund got his 1,000 beds from; but it must be evident that many vacant beds so-called would be those set apart in each hospital for skin diseases, for diphtheria, for cellulitis, or for erysipelas; and it meant that the hospitals did not happen to require beds at the moment for those purposes.

Sir Edmund Currie said there were actually 2,000 beds vacant, and he had deducted 1,000 for the purposes mentioned.

Resuming, Mr. Holland said it might be that there were beds vacant in some hospitals because there were others quite close, and that they were not wanted just there, as, for instance, in the case of Charing Cross.

Sir Henry Burdett said he thought the wording of the resolution was not happy. It committed the Council to impose conditions on its grants without notice to the institutions affected, and it would certainly be held to be an interference with their internal administration. He would suggest that the resolution should read "That notice be given to each institution seeking a grant that the Distribution Committee is instructed that before any hospital or institution receiving a grant from this Fund enters into a contract for the provision of addition accommodation for patients, or any extraordinary expenditure which would increase the cost of maintaining the hospital, it is desirable that the consent or approval of the Council of this Fund should be obtained." That would be a clear notification that extravagant and ill-matured schemes of extension were against the policy of the Fund, and against the interests of the hospitals themselves. The suggestion commended itself especially to the large givers, because they were men of business who were opposed to hasty extravagance. It was essential to have a clear idea not only where to get the money to build, but the money to maintain the additional beds and work. It was certainly lamentable that in the case of St. Mary's, for instance, an extension should stand empty for years; it must entail a charge for maintenance even as an unoccupied building, and it could surely never have been entered on had it been realised that money for maintenance would not be obtainable. The empty beds in the Waterloo Road Hospital were on a somewhat different footing, but he was sure that one reason why the Sunday Fund was not so prosperous as it had been was that the general feeling with regard to building expenditure was hostile to it, and that it had permeated through the congregations to the preachers. An impression was getting abroad that, seeing the hospitals were so extravagant in building, they must have more money than they required for maintenance. It was in the interests of the voluntary system that such an opinion should be proved to be unfounded, and for this reason he was in favour of a resolution being passed inviting the hospitals to scrutinise and limit the outlay on buildings for the future.

Mr. Layton suggested that the wording of the resolution might be "That, in view of the difficulty in raising funds for the hospitals and of the number of beds at present unoccupied, no institution shall enter into a contract for the provision of additional accommodation for patients or any extraordinary expenditure increasing the cost of maintenance without placing the matter before the Council."

Sir Joseph Savory suggested that the representatives of the London hospitals should meet and exchange views on the subject at the Mansion House.

The Rev. C. H. Grundy put forward as an alternative to the resolution and amendments already suggested the following: "That any hospital or institution at present receiving a grant from this Fund be invited to consult the Council of the Fund before incurring further expense in providing additional accommodation for patients."

After further discussion Mr. Hale withdrew his original resolution in favour of that of Mr. Grundy, which he seconded, and which was adopted.

Mr. Pearce Gould suggested that they might also pronounce against any schemes for starting new hospitals unless the Council were consulted, but the Chairman expressed the opinion that they had gone far enough, and the motion was not pressed.

It was mentioned that the £20,000 coming to the Fund under the will of the late Mr. Vokings, of Brighton, was now released; gratuities to the clerks and to the secretary were sanctioned; and votes of thanks brought the proceedings to a close.

